# Stage 1 and 2 Palliation: Comparing Ductal Stenting and Aorto-Pulmonary Shunts in Single Ventricles with Duct-Dependent Pulmonary Blood Flow

**DOI:** 10.1007/s00246-023-03386-5

**Published:** 2024-01-24

**Authors:** Srujan Ganta, Jessica Haley, Howaida El-Said, Brian Lane, Shylah Haldeman, Tara Karamlou, John Moore, Rohit Rao, John J. Nigro

**Affiliations:** 1 Cardiothoracic Surgery, Rady Children’s Hospital + University of California San Diego, 3020 Children’s Way, MC5004, San Diego, CA 92123 USA; 2 Pediatrics, Division of Cardiology, University of California San Diego, San Diego, CA USA; 3 Division of Pediatric Cardiac Surgery, Heart, Vascular, and Thoracic Institute, Cleveland Clinic, Cleveland, OH USA

**Keywords:** Cavo-pulmonary connection, Neurocognitive deficits, Single ventricles, Bi-directional Glenn, PDA stenting, Ductal stenting

## Abstract

Patent ductus arteriosus stenting (PDAS) for ductal-dependent pulmonary blood flow (DDPBF) provides a new paradigm for managing neonates with single ventricles (SV). Currently, sparse data exist regarding outcomes for subsequent palliation. We describe our experience with inter-stage care and stage 2 (S2P) conversion with PDAS in comparison to a prior era of patients who received surgical aorto-pulmonary shunts (APS). Retrospective review of 18 consecutive DDPBF SV patients treated with PDAS between 2016 and 2021 was done and compared with 9 who underwent APS from 2010 to 2016. Patient outcomes and pulmonary artery (PA) growth were analyzed. S2P was completed in all 18 with PDAS with no cardiac arrests and one post-S2P mortality. In the 9 APS patients, there was one cardiac arrest requiring ECMO and one mortality inter-stage. Off cardiopulmonary bypass strategy was utilized in 10/18 in the PDAS and 1/9 in the APS group (*p* = 0.005) at S2P. Shorter ventilation time, earlier PO feeding, and shorter hospital stay were noted in the PDAS group (*p* = 0.01, *p* = 0.006, *p* = 0.03) (S2P). Median Nakata index increase inter-stage was not significant between the PDAS and APS at 94.1 mm^2^/m^2^ versus 71.7 mm^2^/m^2^ (*p* = 0.94). Median change in pulmonary artery symmetry (PAS) was − 0.02 and − 0.24, respectively, which was statistically significant (*p* = 0.008). Neurodevelopmental outcomes were better in the PDAS group compared to the APS group (*p* = 0.02). PDAS provides excellent PA growth, inter-stage survival, progression along multistage single-ventricle palliation, and potentially improved neurodevelopmental outcomes. Most patients can be transitioned through 2 stages of palliation without CPB.

**Supplementary Information:**

The online version contains supplementary material available at 10.1007/s00246-023-03386-5.

## Introduction

Patent ductus arteriosus (PDAS) stenting has evolved as a treatment strategy for patients with ductal-dependent pulmonary blood flow (DDPBF). Our team contributed to the development of this technique in animal studies as well as through clinical practice providing the unique opportunity to understand single-ventricle palliation with this new paradigm [1–3].

Recent studies have compared PDAS to the traditional technique of surgical pulmonary arterial shunts. Some have suggested that PDAS provides the advantage of lower mortality and less need for extracorporeal membrane oxygenation (ECMO) [4]. Others have shown equivalent survival with the benefit (PDAS) of more symmetric pulmonary artery (PA) growth at the expense of higher re-intervention rates [5, 6].

Arterial pulmonary shunts (APS) presently have a mortality rate of 2–12% depending on year of operation in contemporary series and are high mortality risk procedures [7–9]. This mortality risk along with comparable morbidity has generated interest in exploring alternative techniques, such as PDAS.

We have internally reviewed management of single-ventricle (SV) DDPBF with AP shunting and ductal stenting over time at our institution. As a result of this and with advanced interventional skills, our team has successfully altered its practice algorithm. Starting in 2016, we transitioned to a strategy of primary PDAS from surgical shunts based on emerging data suggesting benefits of the PDAS strategy [10, 11]. This transition has provided for a cohort of single-ventricle patients that have had S1P with PDAS and the opportunity to study this strategy on single-ventricle palliation.

SV patients have fragile physiology and are known to be neurodevelopmentally at risk [12, 13]. PA development plays a significant role in subsequent palliation and this has impact on re-intervention rates and health-related quality of life [14]. Presently, there is limited information regarding inter-stage experience and subsequent palliation outcomes for SV patients. Our goal is to review our experience with inter-stage care and stage 2 conversions for DDPBF SV patients who underwent PDAS and compare the overall care pathway to the traditional APS strategy.

## Materials and Methods

### Patients

This retrospective study captured neonates who met inclusion criteria between Jan 2010 and Nov 2021 (single ventricles with DDPBF) at Rady Children’s Hospital San Diego (RCHSD). The study was approved by the institutional review board at RCHSD and the University of California San Diego (UCSD). The study is retrospective and prospective chart review only. A waiver of informed consent and waiver of HIPAA authorization is part of the study IRB. IRB #201333/RCHSD#4652 8/12/2020.

### S1P

Prior to 2017 single-ventricle patients with DDPBF typically received surgical APS. Cardiopulmonary bypass (CPB) was utilized in most cases and cross-clamping (XC) was done when necessary. The APS patients represent ERA 1 of our management strategy. In the year 2016, we began transitioning to PDAS. Our PDAS patients represent ERA 2 in this study.

Surgical shunts used were Gor-Tex™ shunts and typically 3.5 mm or 4 mm in diameter (Supplementary Table 1). Size and placement of shunt was surgeon dependent. The configuration of implantation was a modified BT shunt to the right pulmonary artery or left pulmonary artery or a central shunt. Patients were anti-coagulated with ASA postoperatively until the day of shunt take down.

**Fig. 1:**
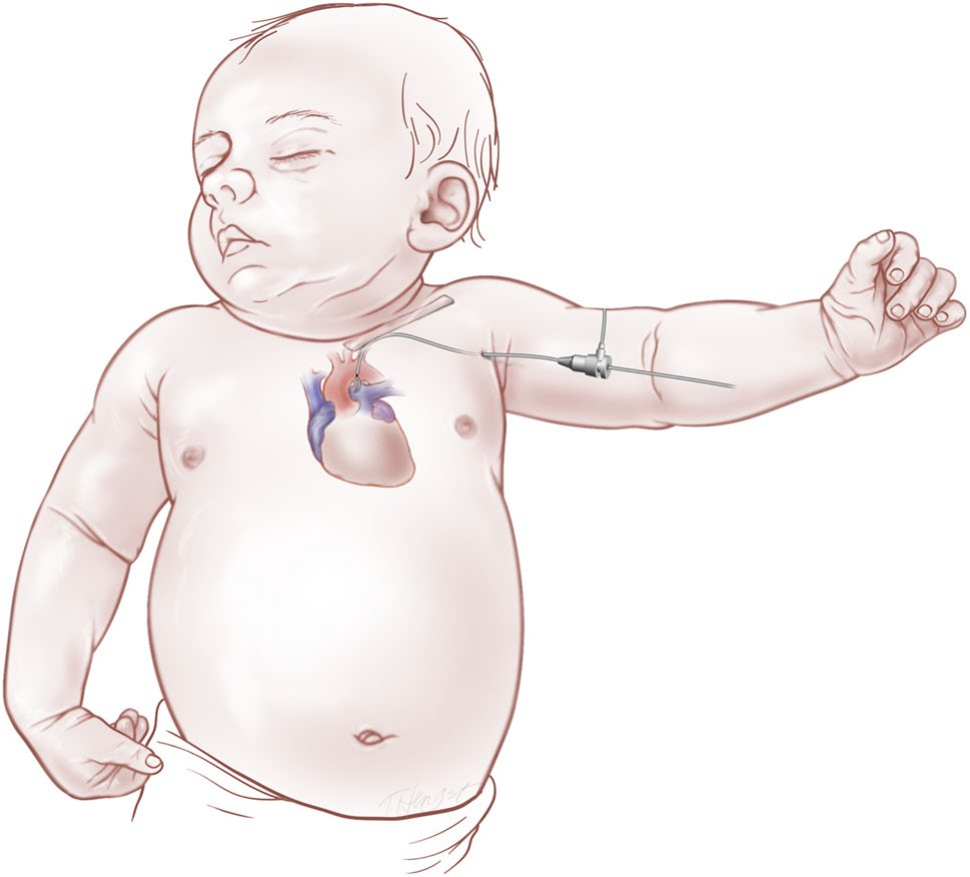
Left axillary approach to ductal stenting. Inset image depicts stent deployment in the PDA

**Fig. 2:**
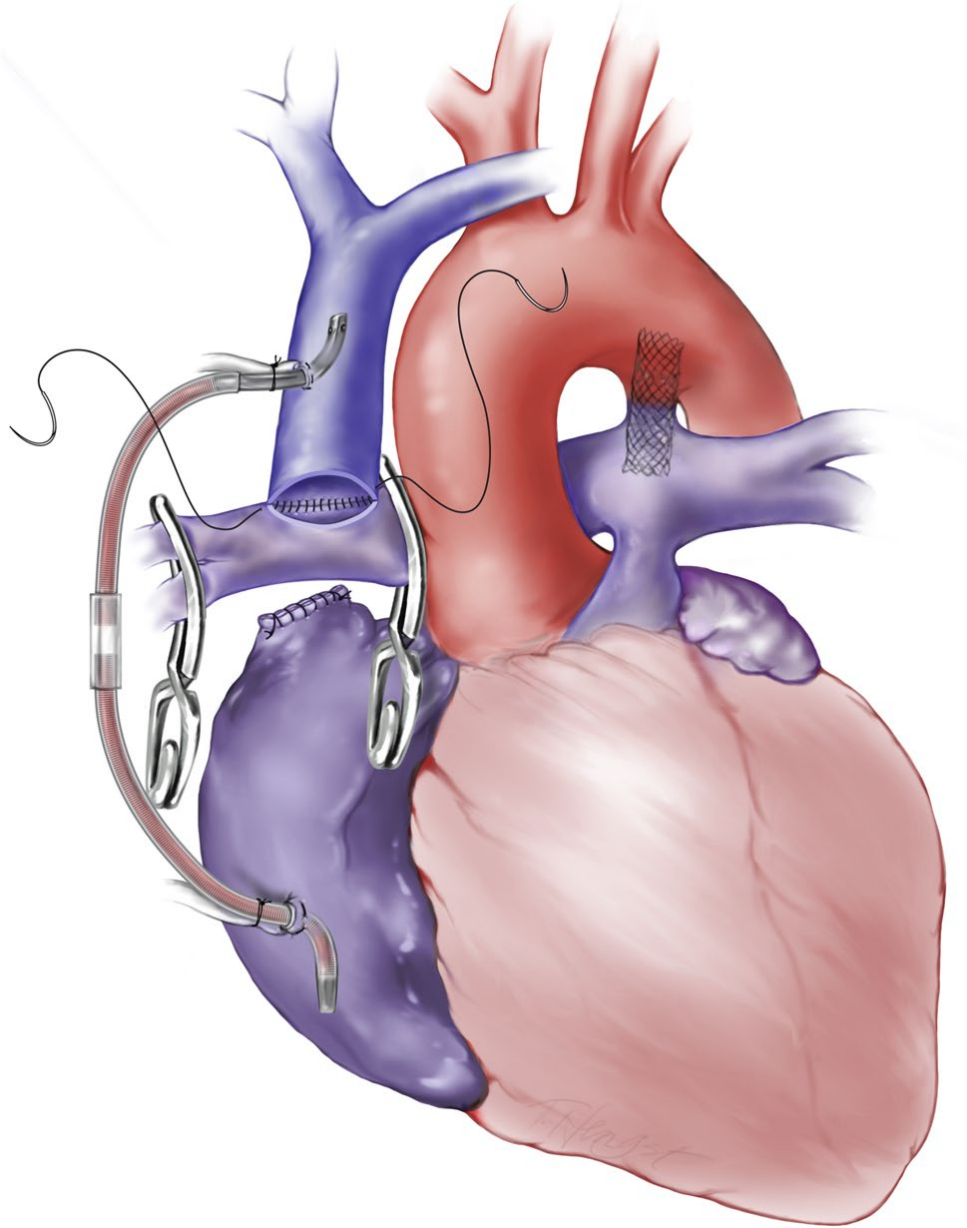
Right-sided Glenn construction with PDAS in the Main pulmonary artery utilizing and SVC-Right atrial shunt

The Onyx drug eluting coronary artery stent (DES) was utilized in patients who underwent PDAS. The access for PDA stent was dictated by the origin of the PDA with the axillary approach being the preferred route due to its direct access to the reverse oriented PDA that arises from the under surface of the arch (Fig. 1). The PDAS may be oriented into the MPA (Fig. 2) or into a proximal branch PA depending on patient anatomy. The more complex the anatomy, the more challenging the PDAS procedure. The risk associated with the more challenging procedures has been mitigated by growing experience and confidence within our team.

After PDAS, patients were transferred to the cardiothoracic intensive care unit and anti-coagulated with ASA + Clopidogrel. Dual anti-platelet therapy was continued until stage 2 and Clopidogrel was stopped 7 days prior to the operation and ASA was continued until the day of surgery.

### Inter-stage

After discharge, patients were followed in a “Home Monitoring Program” where the care algorithm is identical to that of HLHS patients in terms of outpatient surveillance. Cardiac catheterization is done routinely around 3 months of age. Patients were advanced to stage 2 palliation (S2P) when they were 3–5 months of age and roughly 5 kg or sooner if they demonstrated concerning clinical features. Patients typically were assessed for Glenn (S2P) candidacy and had PA pressures measured with this pre-Glenn catheterization.

### S2P (BDG)

APS patients underwent S2P with complete excision of shunt tissue when possible. The shunt causes abnormal changes in the surrounding PA and this is excised completely to prevent abnormal PA growth in future. The residual defect is patched with homograft if necessary to prevent any localized areas of PA stenosis.

PDAS patients underwent S2P with an off-pump strategy when possible by placing a venous shunt from the superior vena cava (SVC) to the right atrium (Fig. 2).

**Fig. 3:**
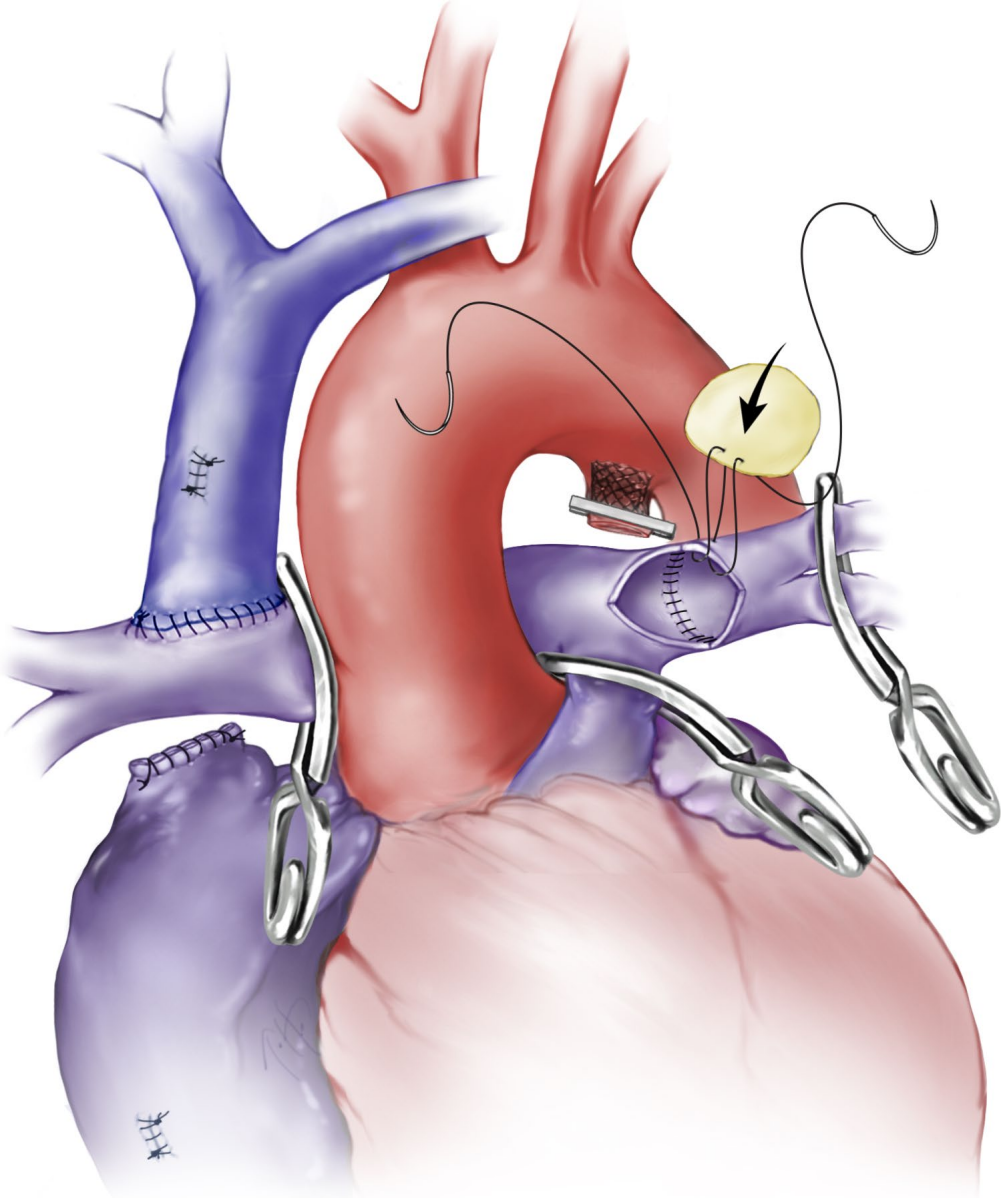
Homograft patch reconstruction of branch pulmonary arteries off cardiopulmonary bypass

After BDG construction in PDAS patients, the ductal stent (DS) is isolated from the branch PA’s using neuro-clips. The DS is occluded with a metal hema-clip and then cut and pulled out of the PA (Supplementary Fig. 1). After stent removal, the PA can be patched to prevent a focal stenosis. (Supplementary Fig. 2). We also utilize an SVC plasty technique in which the SVC is splayed open and sewn onto an extended pulmonary arteriotomy. Segmental resection was performed for ductal coarctations with primary back-wall reconstruction and anterior homograft patch augmentation (Fig. 3). Standard post-operative monitoring and assessment is utilized after S2P.

### Post-S2P

Patients received routine catheterization 2- 4-month post-S2P for hemodynamic monitoring and anatomic surveillance. This is an institutional preference and identifies any potential kinks or flow limiting lesions. During this catheterization aorto-pulmonary collaterals are also closed to ensure the ventricle is unloaded and has become part of our routine single-ventricle surveillance.

Neurodevelopmental (ND) assessments included but were not limited to gross motor function, interactive skills, feeding, language ability, and visual responsiveness completed by neonatologists and cardiologists. Recently we transitioned to the Ages and Stages Questionnaire (ASQ) to standardize our ND follow-up with a validated tool [15]. Since part of our ND data consists of the ASQ methodology and prior assessments were of the aforementioned criteria, we organized our assessments into broad categories that allowed us to merge the collected data and report it in a simplified way. Patients were categorized as having no ND impairments or identified ND deficits.

### Statistics

Statistical analysis was performed using statistical software tool R version 4.04. Descriptive statistics were calculated for all variables of interest and were reported as medians and interquartile (IQR1–IQR3) ranges. As our variables of interest deviated from normality, we used Wilcoxon-paired signed ranks tests for our temporally related matched data and Mann–Whitney *U* tests when comparing different treatments as unmatched groups. Statistical significance was considered as *p* < 0.05.

Weight for age *z*-scores (WAZ) Centers for Disease Control (CDC) and Prevention 2000 were used as a growth parameter to facilitate comparison during palliation stages. Pulmonary artery (PA) architecture was quantified using Nakata indexes and pulmonary artery symmetry scores (PAS) at each stage of palliation. PAS was calculated by dividing the diameter of the smaller branch PA by the diameter of the larger branch PA (measured at the takeoff of the upper lobe branch) and this is a unitless value. Smaller values are indicative of less symmetric PA’s. Calculations were based on measurements from CT scans and cardiac catheterization.

## Results

### Demographics

In the study period, 18 patients received PDAS and 9 received APS. Demographics are listed in Table 1. There was no significant difference between PDAS and APS in terms of gestational age, birth weight, weight at S1P, or weight for age *z*-scores (WAZ). Cardiac morphologies are displayed in Supplementary Fig. 3. All patients had restricted pulmonary blood flow or were ductal dependent and were classified into one of seven categories: Pulmonary atresia intact ventricular septum (PA-IVS) (7), Tricuspid atresia (4), Heterotaxy variants (10), DILV/PA (3), Unbalanced AVC (1), Ebstein’s (1), and L-TGA/DORV (1).

**Table 1:** Patient demographics and pulmonary architecture

		PDAS (*n* = 18)		APS (*n* = 9)	*p* value
Female		8		2	0.28
Age					
Gestational age (weeks)		39 (37–39)		39 (33–40)	0.83
Age at first procedure (days)		8 (6–18)		11 (9–81)	0.18
Age at stage 2 (months)		4.7 (3.6–6.5)		7.8 (4.4–10.6)	0.04
Inter-stage time (months)		4.2 (3.3–6.3)		6.6 (3.7–7.9)	0.01
Weight					
Birth weight (kg)\[WAZ]		3.2 (2.8–3.4)\[− 0.43]		3.12 (1.8–3.3)\− 0.73	0.20
Weight at stage 1 (kg)\[WAZ]		3.3(3.0–3.5)\[− 1.2]		3.4 (3.2–3.9)\− 1.07	0.92
Weight at stage 2 (kg)\[WAZ]		6.1 (5.2–6.8)\[− 1.2]		6.6 (6.1–7.5)\− 1.7	0.11
Weight at F/U\WAZ		10.7 (8.8–13.5)\[− 0.2]		13.3 (7.4–19)\− 1.47	0.04
Pulmonary architecture					
Nakata index S1P, median		121 (99–149)		143 (84–209)	0.62
Nakata index S2P, median		229 (191–264)		257 (159–330)	0.72
Change in Nakata Index between stages (mm^2^/m^2^)		+ 94 (64–146)		+ 71.6 (52–200)	0.94
PA symmetry score S1P		0.91 (0.81–0.96)		0.88 (0.8–0.99)	0.82
PA symmetry score S2P		0.86 (0.76–0.94)		0.62 (0.44–0.78)	0.002
Change in PAS between stages		− 0.02 (− 0.16 to 0.03)		− 0.24 (− 0.37 to − 0.18)	0.008
Nakata index at Follow-up, (14/25 pts)		187(179–207)		223.5 (153–304)	0.78
PA symmetry score at Follow-up (14/25 pts)		0.88 (0.73–0.98)		0.89 (0.79–0.95)	> 0.99
Ventilation					
Duration ventilation S1P hours		13.8 (3.4–20.3)		95 (70–212)	< 0.001
Duration ventilation S2P hours		7.1 (4.3–10.1)		11.6 (10.1–47.1)	0.01
Feeding status S2P DC		17		8	
PO feeding		15/17 (88%)		4/8	0.006
PO + NG feeding		0		0	0.52
G-tube feeding		2		4/8	0.28
Mortality		1/17 (neurologic injury, post-PDAS)		1/9 PV stenosis (Inter-stage)	0.53
LOS					
Total hospital LOS S1P, d		19.5 (13–52)		30 (17.5–107)	0.08
LOS post-S1P, d		10 (7–45)		21 (14–76)	0.02
Total hospital LOS S2P, d		7 (5–12)		13 (8.5–85)	0.03
LOS post-S2P, d		7 (5–10)		9 (7–17)	0.11
Follow-up post-BDG					
Follow-up time (months)		16.5 (5.4–33.6)		49 (9.7–82)	0.13
Time to Re-intervention post-S2P, days		83 (58–272)		24.5	0.12
Re-intervention post-BDG		3/17		2/8	0.71

Values are Median (IQR interquartile range)

*PDAS* patent ductus arteriosus stenting, *G-tub*e gastrostomy tube, *BDG* bi-directional Glenn, *LOS* length of stay, *DC* discharge, *PO* Per Os, *NG* Naso-gastric, SpO2 arterial saturations, SSI superficial sternal infection, *TPG* trans-pulmonary gradient, *S1P* stage 1 Palliation, *S2P* stage 2 Palliation, WAZ weight for age z-scores, PAS pulmonary artery symmetry score

### Stage 1 Palliation/Initial Palliation

PDAS survival to S2P was 18/18 (100%) with no cardiac arrests and no ECMO use. All APS patients survived initial procedure and there was a single mortality (1/9) 11% inter-stage secondary to progressive pulmonary vein stenosis (Table 1).

PDAS patients did not require CPB, while APS utilized CPB in 8/9 (89%) cases.

One APS patient arrested on post-operative day (POD) #1 for unknown reasons and required temporary ECMO support.

Of PDAS deployments, 17/18 (94%) were successful with 1 conversion to an mBT shunt (Table 2). Initial stent deployment did not cover the entire duct and there were concerns over re-crossing this area. An mBT shunt was then placed under non-emergent conditions while on a prostaglandin infusion.

**Table 2:** Interventions and operative data

	PDAS	APS	*p* value
Patients	18	9	
PDAS conversions to mBT	1/18	–	
Inter-stage re-interventions	10/18	5/9	0.93
Stent fracture	2/10	–	
Intimal buildup/stenosis	5/10	–	
Time to re-intervention, months	2.71 (1.55–3.44)	1.54 (0.76–1.87)	0.03
Operative data stage 1			
CPB			
Off-pump	18	1/9	< 0.001
On-pump	0	8/9 (4 required XC)	< 0.001
CPB	–	117 (66–145)	
XC (min)	–	27 (15–73)	
Operative data stage 2	(1 patient did not progress)		
CPB			
Off-pump	10/17	1	0.005
On-pump	7/17	7	0.01
CPB (min)	85 (34.5–145.5)	122 (91–162)	0.16
XC (min)	0 (0–0)	43 (13–114)	0.05

Values are Median (IQR interquartile range)

*PDAS* patent ductus arteriosus stenting, *CPB* cardiopulmonary bypass, *XC* cross-clamp, *mBT* modified Blalock–Taussig shunt

Nakata index and pulmonary artery symmetry (PAS) score at S1P for PDAS was 121 mm^2^/m^2^ (99–149) and 0.91 (0.81–0.96), respectively, while APS demonstrated values of 143 mm^2^/m^2^ (84–209) and 0.88 (0.80–0.99), respectively, which were not significantly different between the 2 groups (Table 1).

PDAS post-procedure ventilation time was 13.4 (5.7–20) h and the difference between ERAs was significant as median time ventilated in APS was 95 (70–212) hours (*p* < 0.001) (Table 1).

**Fig. 4:**
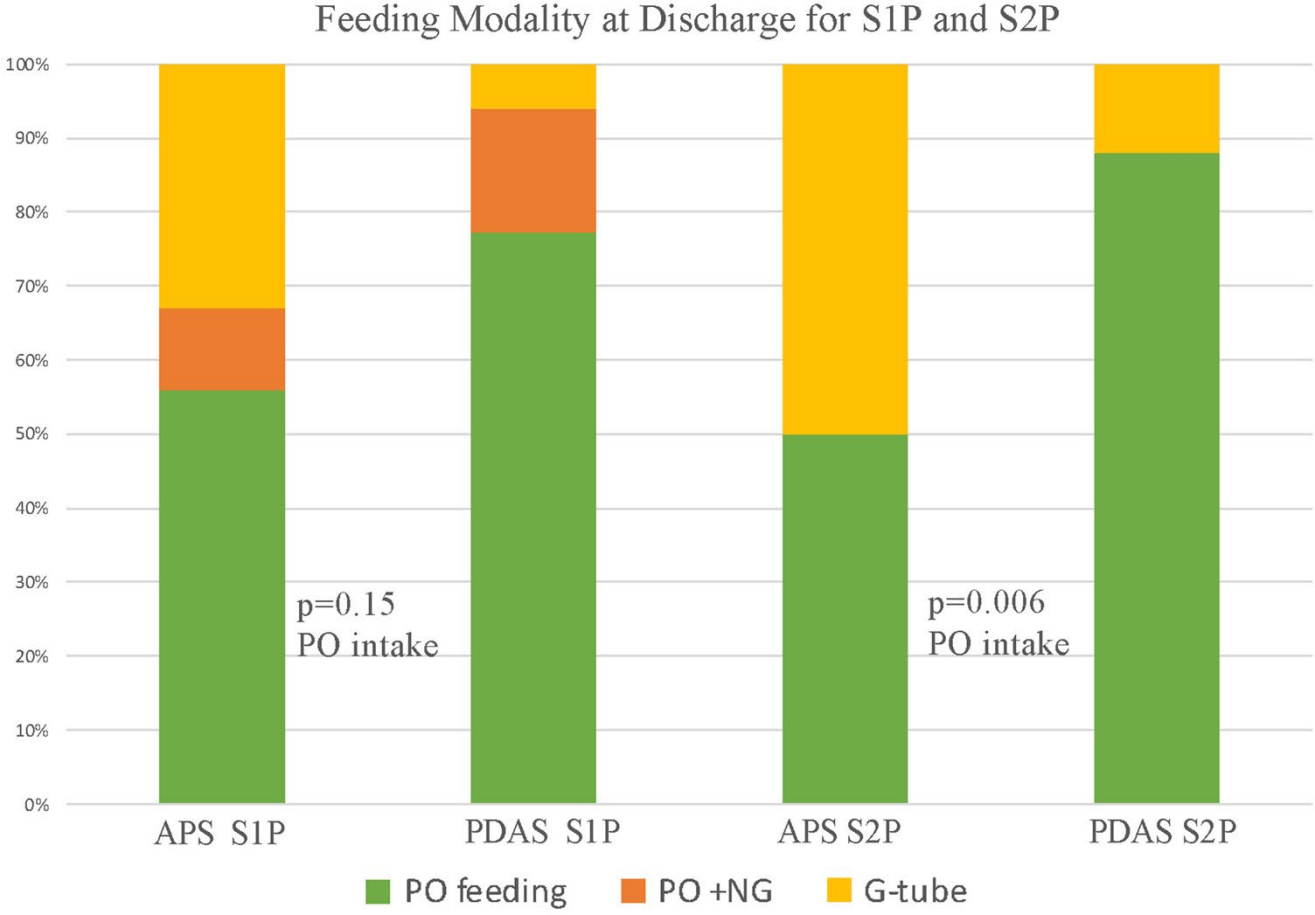
Feeding strategy post-S1P for PDAS and APS patients

PDAS patients were discharged home PO feeding in 14/18 (78%) of cases, 3/18 (17%) were PO feeding with NG supplementation and a single gastrostomy tube was inserted 1/18 (6%) (Fig. 4). With APS, 5/9 (56%) were PO feeding, 1/9 (11%) were PO with NG supplementation, and 3/9 (33%) had gastrostomy tubes inserted. The difference did not reach significance.

There was a significantly lower post-procedure hospital length of stay with PDAS at a median of 10 (7–45) days with APS patients staying 21 (14–76) days (*p* = 0.02).

### Inter-stage

There were no inter-stage arrests, ECMO cannulations or mortalities with PDAS. APS patients had a single 1/9 (11%) mortality from progressive pulmonary vein stenosis.

PDAS patients had 10/18 (56%) re-interventions at a median of 2.71 (1.55–3.44) months.

For APS patients, 55% (5/9) underwent inter-stage re-interventions at a median of 1.54 (0.76–1.87) months. Re-intervention rates were not significantly different between PDAS and APS but time to re-intervention was significantly lower with APS (*p* = 0.03).

Of the 5 patients that had re-intervention post-APS, 2 patients received stenting of the shunt or branch PA and 2 required ballooning of the shunt or PA. One patient required balloon dilations for progressive pulmonary vein stenosis as well as upsizing of the central shunt and was the single mortality.

For PDAS patients, 6 patients had stenosis on follow-up catheterization requiring stenting. Stent fractures were found in 2 and these were also re-stented. One patient received ballooning of a branch PA.

One patient was deemed a non-candidate for advancement to BDG due to progressive pulmonary vein stenosis. This patient had a surgical shunt created while undergoing pulmonary vein un-roofing and an atrial septectomy. This patient remains shunted and has not progressed to S2P.

Pre-Glenn catheterization was performed in 7 of the APS patients with a median mPAP of 14 (11–15) mmHg. Of the 2 who did not have a catheterization, one had a post-Glenn catheterization mPAP of 10 mmHg.

Pre-Glenn catheterization was done in 16/18 of the PDAS patients with a median mPAP of 10.5 (9–14) mmHg. The 2 patients who did not have catheterization done had post-Glenn catheterization and both had an mPAP of 10 mmHg.

### Stage 2 Palliation

PDAS S2P survival was 94% (16/17) with a mortality occurring from progressive perinatal neurologic injury. Retrospective MRI SVC flow analysis in this patient demonstrated flow of 0.16 L/min and 0.67 L/min/m^2^ prior to BDG.

Median PDAS age at stage 2 was 4.7 (3.6–6.5) months. Median weight at S2P was 6.1 (5.2–6.8) kg representing a median growth of + 2.8 (2.0–3.4) kg inter-stage. WAZ remained unchanged at − 1.2 between stages.

APS SP2 survival was 8/8 (100%). Median age and weight were 7.8 (4.4–10.6) months and 6.6 (6.1–7.7) kg. This represented a median growth of + 3.6 (2.3–4) kg inter-stage. WAZ at stage 2 was − 1.7 in the APS group.

**Fig. 5:**
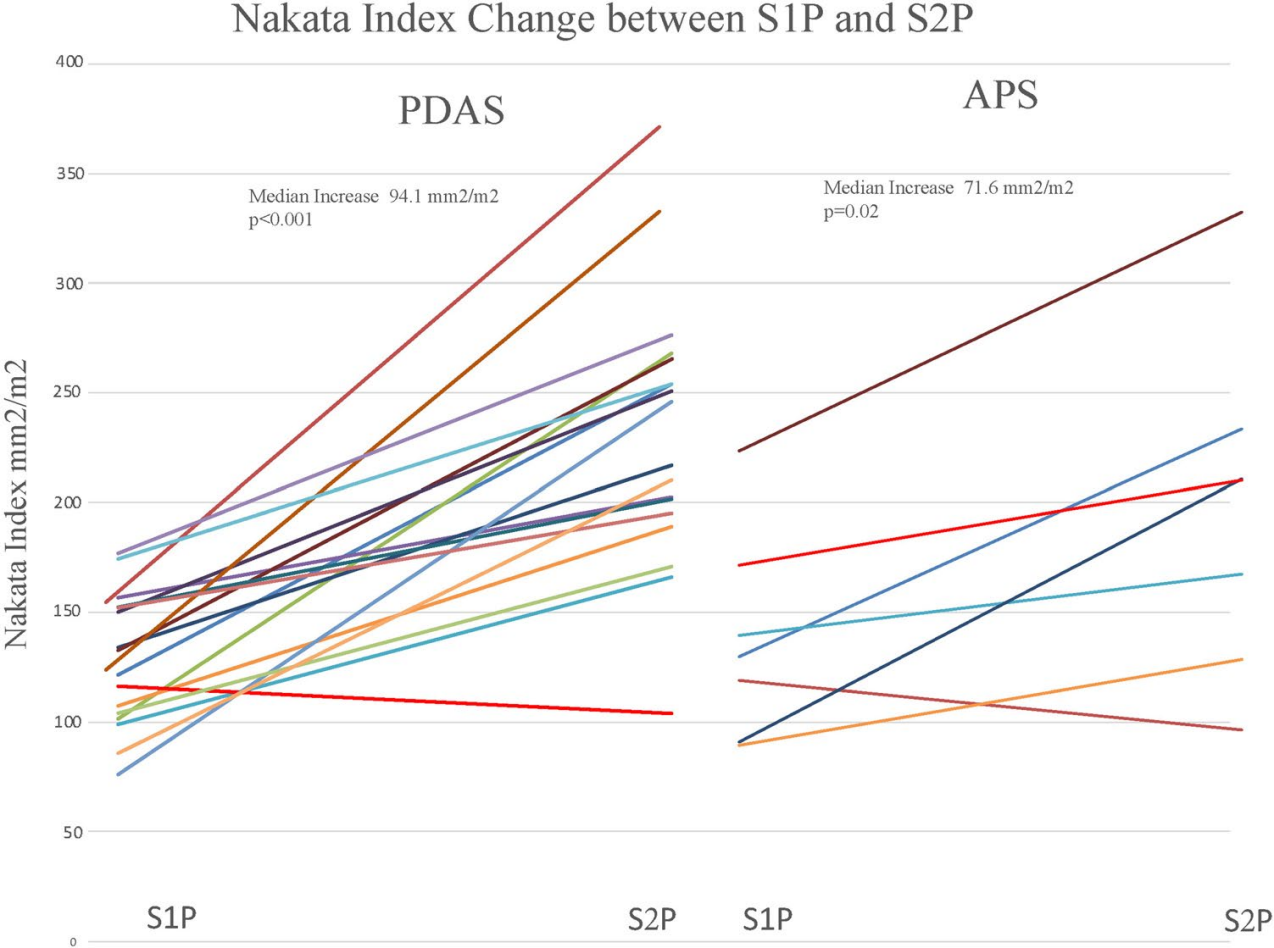
Nakata indexes for patients at S1P and S2P for both PDAS and APS demonstrating pulmonary artery growth interstage. The listed p-values indicate the growth in both groups of patients was significant inter-stage. The difference in growth between PDAS and APS however was not significant as listed in our data tables

PDAS patients Nakata index increased from 121 (S1P) to 229 mm^2^/m^2^ (S2P) representing a median increase of 94.1 mm^2^/m^2^ (64–146) (Fig. 5). PAS changed from 0.91 to 0.86 with a median decrease of − 0.02 (− 0.16 to 0.03). APS patient’s median Nakata index increased from 143 (S1P) to 257 mm^2^/m^2^ (S2P) representing a change of 71.7 (52–200) mm^2^/m^2^. Median PAS changed from 0.88 to 0.62 representing a median decrease of − 0.24 (− 0.37 to − 0.18).

Pulmonary artery growth as evidenced in Nakata index did not reach significance when comparing PDAS and APS (*p* = 0.94). The change in PAS score was significant between PDAS and APS (*p* = 0.008). PDAS maintained pulmonary artery symmetry, while APS led to more asymmetric branch PA growth.

Of 18 patients with PDAS, CPB was required in 7 patients and the remaining 11 (61%) had their S2P constructed off CPB with a venous shunt. Median CPB time was 85 (35–150) min in this group and none required aortic cross-clamping. More PDAS patients compared to APS had their S2P done off-pump (*p* = 0.005).

In APS patients at S2P, all but one required CPB with a median time of 122 (91–162) min and a median XC time of 43 (13–114) min.

Patients received augmentation of the branch PAs or the main PA in 63% (5/8) of cases with APS and 53% (9/17) cases with PDAS. Of patients requiring PA branch augmentation after PDAS, 5/9 of the branch augmentations were done off CPB (Table 3).

**Table 3:** Miscellaneous data

	PDAS	APS	*p* value
Patients	17	8	
PA branch augmentation	9/17	5/8	0.68
PA flap advancement angioplasty	1/17	–	–
Homograft patch angioplasty	3/17	5/8	–
Autologous pericardium angioplasty	1/17	–	–
BDG SVC angioplasty	4/17	–	–
PA branch augmentation on CPB	4/17	5/8	–
PA branch augmentation off CPB	5/17	0	–
Neurodevelopmental status at last follow-up	17	5	–
Normal development	11/17 (64%)	1/5(20%)	0.02
Developmental delay	6/17 (35%)	4/5 (80%)	0.09
No data	–	3	

Values are Median (IQR interquartile range)

*CPB* cardiopulmonary bypass, *PA* pulmonary artery, *BDG* bi-directional Glenn, *SVC* superior vena cava, *RPA* right pulmonary artery, *ND* neurodevelopment

Ventilation time post-S2P for PDAS was shorter compared to APS 7.1 (4–10) h and 11.6 (10–47) h, respectively (*p* = 0.01). PDAS patients were orally feeding in 94% (16/17) of cases, while 50% (4/8) APS patients were orally feeding (*p* = 0.006).

### Outcomes Post-Stage 2 Palliation

Follow-up post-S2P was completed for all patients with a median follow-up time of 16.5 months (5.4–33.6) for PDAS patients and 49 months (9.7–8.2) for our APS patients.

In terms of post-S2P catheterizations, PDAS patient’s median Nakata index was 186.5 mm^2^/m^2^ (179–207) and APS patients was 223.5 mm^2^/m^2^ (152–304) and this was not significant (*p* = 0.94).

PAS for PDAS and APS were 0.88 (0.73–0.98) and 0.89 (0.80–0.95), respectively. Re-interventions have been performed in both groups and are listed in Table 2.

The number of patients who had normal ND outcomes was 64% (11/17) in PDAS and 20% (1/5) with APS (*p* = 0.02).

**Fig. 6:**
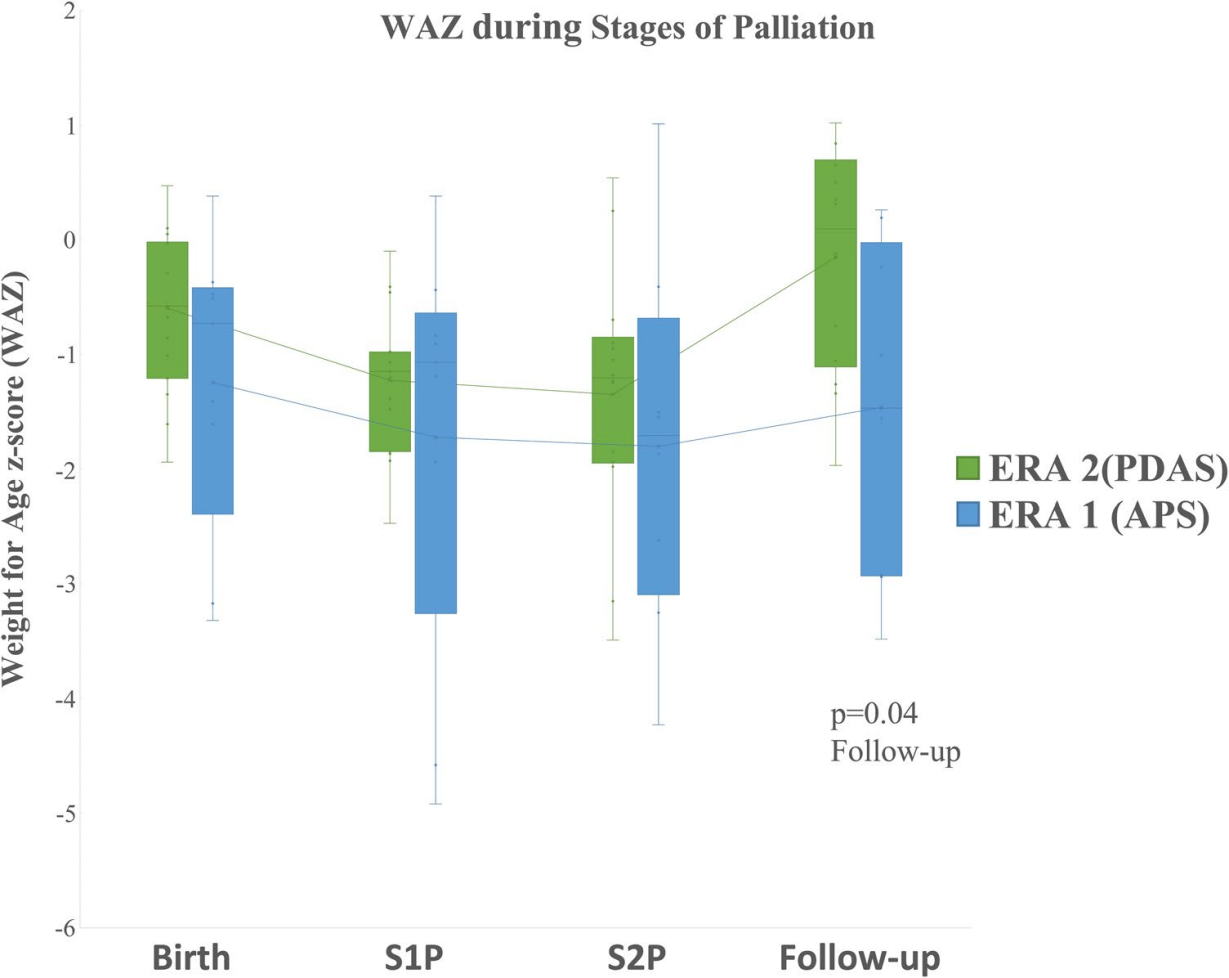
Change in WAZ (weight for age *z*-score) from birth to present follow-up. Superimposed line graph represents mean WAZ as followed through stages. The upper and lower limits of the green boxes represent the first (Q1) and third (Q3) quartiles. The line in the box represents medians, while the whiskers represent minimum and maximum values. There was a significant difference between WAZ for the 2 groups at follow-up

The difference between WAZ at follow-up was significant between PDAS (− 0.2) and APS (− 1.47) (*p* = 0.04). Overall growth results are graphically depicted in Fig. 6.

## Comment

This study reports PDAS in palliated single-ventricle patients with DDPBF and compared this contemporary strategy with surgical APS.

### PDAS Technique

PDAS is an interventional technique and this technology’s deployment is analogous to the deployment of any surgical procedure. There is an associated learning curve and the development of experience and confidence over time will allow for more complex PDAS deployments. With that said, stenting of complex anatomy has the potential to fail requiring urgent conversion to a surgical BT shunt. Depending on the urgency of conversion, patients may be unstable which can increase the overall risk of a surgical procedure and this possibility should be considered during the initial decision-making process. Although this has not been the case in our patient population, it is conceivable that high-risk ductal anatomy in some patients is best managed with a surgical shunt as a first-stage procedure.

### Neurodevelopmental

SV patients are at risk for poor ND outcomes and based on IQ tests 25% to 33% are moderately impaired (IQ < 85) and 10–20% severely impaired (IQ < 70) [12, 13] as children and adolescents. The PDAS patients in this study were more likely to have normal ND assessments in follow-up than the APS patients. It is important to note that ND data collection changed over time as outlined in our methods which limits the strength of our conclusions. Recognizing this, the statistically significant number of PDAS patients with normal ND outcomes in our study raises the question of whether SV patients may be able to achieve better ND outcomes than reported in contemporary literature [12, 13].

This will need to be investigated quantitatively with ASQ scores moving forward to answer this question. Our ND findings are unique and will hopefully lead to further investigation in these patients.

### Feeding

Contemporary reports demonstrate the importance of feeding and weight gain in optimizing care for our patients [16, 17]. In a study specific to DDPBF comparing BTS to PDAS, patients were more likely to be PO feeding overall with PDAS, but only ½ of SV patients were PO feeding after initial palliation [18]. Our rates are similar with APS where half of patients after S2P were G-tube fed but our rate of PO feeding was significantly higher with PDAS as 71% after S1P and 88 percent after S2P were PO fed. This is in part from prioritizing oral feeding with advanced pre- and post-procedural therapies. This effort has been made as we recognize non-oral feeding strategies have been associated with neurodevelopment costs [19]. It is possible that the higher rates of oral feeding are in part responsible for the superior ND outcomes we have achieved with PDAS.

### Growth and *z*-scores

Median WAZ for patients were low at birth and declined during SV palliation for both groups of patients but increased post-S2P. Plotted *z*-scores revealed a trend toward normal growth by last follow-up in our PDAS patients and this reached statistical significance when compared to our APS patients.

In contemporary reports, inter-stage growth is associated with survival in S1P in patients who received Norwood operations [20]. Of particular note, there was no decrease in WAZ which has been associated with late mortality in congenital cardiac surgery [21]. The return to near normal WAZ by 16-month follow-up in our PDAS patients suggests adequate cardiac output post-S2P and is re-assuring in this cohort.

### Pulmonary Artery Growth

PA growth has been studied post-PDAS and compared to APS using Nakata indexes to quantify PA development [5, 6, 22]. This is important as pulmonary artery size and architecture is associated with favorable single-ventricle palliation [14]. PAS was superior for the PDAS cohort between the 2 groups and statistically significant with a noted decline for APS patients inter-stage. It is possible that symmetric PA growth will manifest in lower PA re-interventions over time and better SV preparedness.

### Cardiopulmonary Bypass

Variants of off-pump BDG construction have been in use since the 1950s [23, 24]. Our off-pump BDG technique uses angled cannulas in the SVC and right atrium, connected, creating a shunt. To ensure adequate SVC decompression and optimal neuro-protection, we monitor arterial oxygen saturation, SVC pressure, NIRS, ECG, and arterial pressure closely. The logic driving off-pump BDG utilization is to optimize post-operative BDG physiology and possibly attenuate bypass-associated lung injury. Although controversial, this may facilitate early extubation and may have contributed to our observed ventilatory time differences. We hope to further report on this as our experience grows with our care pathway and the use of off-pump BDG.

### Mortality

The mortality post-APS was secondary to significant pulmonary vein stenosis. The mortality post-PDAS was from perinatal neurologic injury consisting of intracranial hemorrhage. The patient was discharged home after S1P but re-admitted for low saturations and care was ultimately withdrawn post-S2P due to severe neurologic injury.

Retrospective review of an MRI with flow calculations done in the weeks preceding S2P in this PDAS patient yielded both SVC and indexed flows reported in our results.

Our patient had low flows and this provides insight as a subsequent report indicated SVC flows < 0.5 L/min and 1.6 L/min/m^2^ are associated with Glenn failure [25, 26]. This case highlights how PDAS can provide for palliation in the most brittle patients who would normally be considered non-operative candidates.

## Study Limitations

This is a retrospective single-center study and subject to decision-making that is center specific which may introduce institutional bias. The majority of our PDAS patients were in the latter half of our study as we had shifted our primary strategy in 2017. Anatomic lesions in both groups were heterogeneous which may introduce a potential source of error. The study period was over 11 years and during this time there was an evolution of both ideology and clinical expertise at our center in both the operating room and post-operative care which may introduce an ERA effect. The development of our ICU and increased resource allocation to our home monitoring program are the primary contributors to this effect. This may favor patients in the later half of our study as they have the benefit of experience and evolving care at our institution. Our neurodevelopmental data were heterogeneous and derived from 2 different assessment tools as our program evolved limiting its ability to quantify deficits beyond the presence or absence of developmental delay. ASQ data will quantify and qualify the ND differences in future. Also, the sample size is not large consisting of 27 SV patients which will limit study power (increasing likelihood of type II error).

## Conclusion

PDAS has provided attractive results such as decreased ventilation time, superior PAS, higher rates of oral feeding, and superior somatic growth and suggest a potential improvement in neurodevelopmental outcomes compared to APS. PDAS has also provided excellent pulmonary artery growth, pulmonary artery symmetry and post-Glenn physiology. As a result, PDAS has changed SV palliation paradigms for patients with DDPBF at our center. The majority of these patients utilizing PDAS can be transitioned through 2 stages of palliation without CPB.

### Supplementary Information

Below is the link to the electronic supplementary material.

Supplementary file1 **Supplementary Figure 1** The aortic side of the PDA stent has been clipped and the stent transected. The pulmonary artery side of the stent is being pulled out of the main pulmonary artery. (TIF 5516 KB)

Supplementary file2 **Supplementary Figure 2** The defect created by removing the ductal stent from the main pulmonary artery has been corrected with the addition of a homograft patch. (TIF 9811 KB)

Supplementary file3 **Supplementary Figure 3** Cardiac Morphologies for PDAS and APS groups. (TIFF 145 KB)

Supplementary file4 (DOCX 14 KB)

## Abbreviations

APS: Aorto-pulmonary shunts
CVP: Central venous pressure
ND: Neurodevelopment
CPB: Cardiopulmonary bypass
S1P: Stage 1 palliation (ductal stenting)
S2P: Stage 2 palliation (Glenn/BDG)
ECMO: Extracorporeal membrane oxygenation
BDG: Bi-directional Glenn
PAS: Pulmonary artery symmetry score
PDAS: Patent ductus arteriosus stenting
SV: Single ventricle
DDPBF: Ductal-dependent pulmonary blood flow
mBT: Modified Blalock–Taussig shunt
WAZ: Weight for age *z*-score
DS: Ductal stent
PA: Pulmonary artery
mPAP: Mean pulmonary artery pressure
