# Supplementary figures and images for: Stage 1 and 2 Palliation: Comparing Ductal Stenting and Aorto-Pulmonary Shunts in Single Ventricles with Duct-Dependent Pulmonary Blood Flow
Source: Pediatr Cardiol. 2024 Jan 24;45(3):471–82. doi: 10.1007/s00246-023-03386-5 (PMC10891206; doi:10.1007/s00246-023-03386-5)

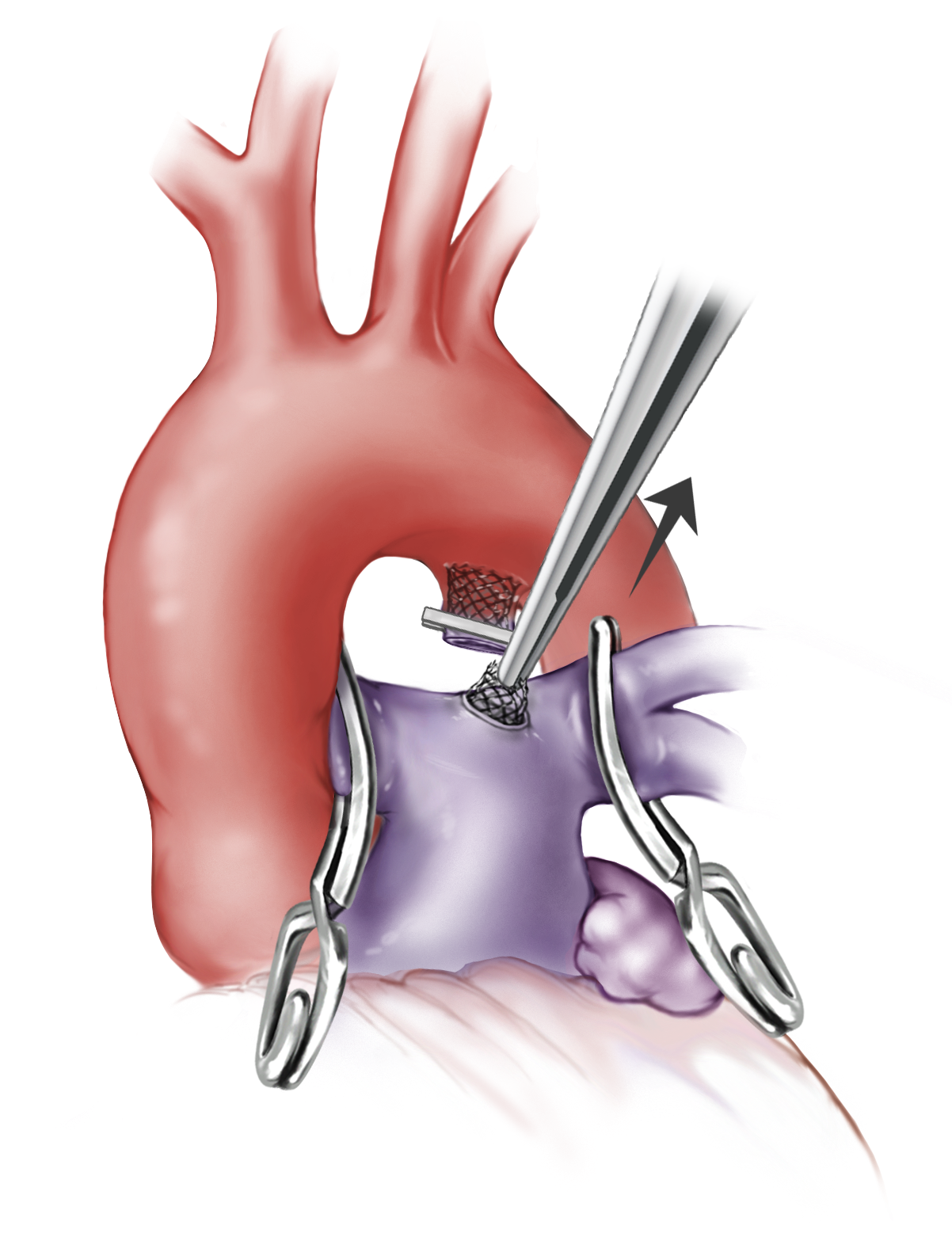

Supplement: Supplementary file 1 — Supplementary file1 Supplementary Figure 1 The aortic side of the PDA stent has been clipped and the stent transected. The pulmonary artery side of the stent is being pulled out of the main pulmonary artery. (TIF 5516 KB) [file 246_2023_3386_MOESM1_ESM.tif]

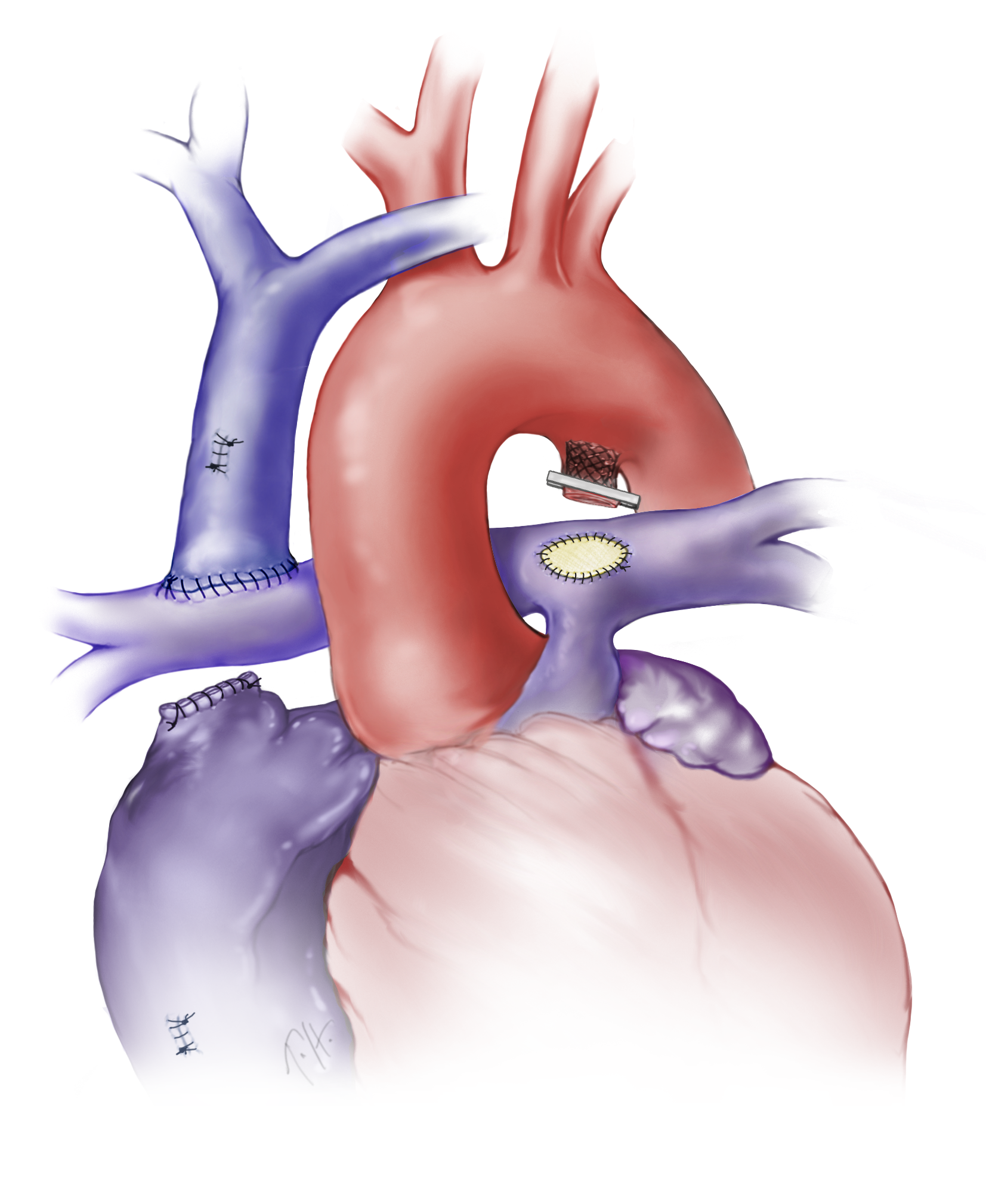

Supplement: Supplementary file 2 — Supplementary file2 Supplementary Figure 2 The defect created by removing the ductal stent from the main pulmonary artery has been corrected with the addition of a homograft patch. (TIF 9811 KB) [file 246_2023_3386_MOESM2_ESM.tif]

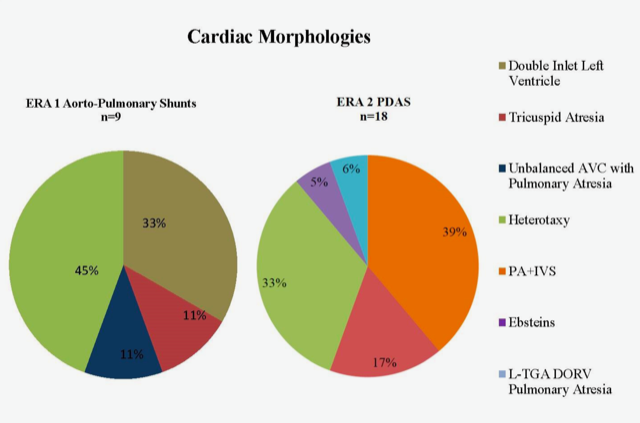

Supplement: Supplementary file 3 — Supplementary file3 Supplementary Figure 3 Cardiac Morphologies for PDAS and APS groups. (TIFF 145 KB) [file 246_2023_3386_MOESM3_ESM.tiff]
