# Supplementary material for: Stage 1 and 2 Palliation: Comparing Ductal Stenting and Aorto-Pulmonary Shunts in Single Ventricles with Duct-Dependent Pulmonary Blood Flow
Source: Pediatr Cardiol. 2024 Jan 24;45(3):471–82. doi: 10.1007/s00246-023-03386-5 (PMC10891206; doi:10.1007/s00246-023-03386-5)
Supplement: Supplementary file 4 — Supplementary file4 (DOCX 14 KB) [file 246_2023_3386_MOESM4_ESM.docx]

| Supplementary Table 1 | | |  |
| --- | --- | --- | --- |
| **Aorto-Pulmonary Shunt Patients** | | | |
| Patient | Shunt Size(mm) | **Type** | Reconstruction Technique |
| 1 | 4 | Central | none |
| 2 | 3.5 | Right-mBT | MPA, RPA, LPA |
| 3 | 4 | central | none |
| 4 | 4 | Right-mBT | MPA, RPA, LPA |
| 5 | 3.3 | Right-mBT | none |
| 6 | 4 | Central | none |
| 7 | 4 | Left-mBT | none |
| 8 | 4 | Central | MPA |
| 9 | 4 | Central | none |

mBT=modified BT shunt; MPA=main pulmonary artery;

RPA=right pulmonary artery;LPA=left pulmonary artery;
